# Case Report: Prenatal and Postnatal Management for Fetal Bronchogenic Cysts During the COVID-19 Pandemic

**DOI:** 10.3389/fped.2021.675883

**Published:** 2021-07-07

**Authors:** Lin Cheng, Jie Duan, Mei Wang, Dan Lu, Huan Li, Jianhong Ma, Juan Liu, Cheng Wang, Yuanzhen Zhang

**Affiliations:** ^1^Department of Obstetrics and Gynecology, Zhongnan Hospital of Wuhan University, Wuhan, China; ^2^Hubei Clinical Research Center for Prenatal Diagnosis and Birth Health, Wuhan, China; ^3^Wuhan Clinical Research Center for Reproductive Science and Birth Health, Wuhan, China; ^4^Department of Ultrasound Imaging, Zhongnan Hospital of Wuhan University, Wuhan, China; ^5^Department of Radiology, Zhongnan Hospital of Wuhan University, Wuhan, China

**Keywords:** bronchogenic cyst, prenatal and postnatal management, MRI, multidisciplinary approaches, COVID-19 pandemic

## Abstract

**Background:** A fetal bronchogenic cyst (BC) is a rare congenital anomaly with an incidence of 0.147–0.238‰. The coronavirus disease 2019 (COVID-19) pandemic, as a particular situation, hindered pregnant women from receiving periodic prenatal checkups.

**Case Description:** Until 34^+6^ weeks of gestation, a fetal case of the intrathoracic cyst was found by ultrasound examination. Further, MRI examination confirmed the diagnosis of the congenital mediastinal cystic lesion, probably a BC. Genetic testing was not conducted due to the COVID-19 pandemic. At 38^+5^ weeks of gestation with maternal COVID-19 testing negative, a live girl was delivered by cesarean section. Five months later, the child underwent bronchocystectomy, and the postoperative pathological lesions confirmed a (right upper mediastinum) BC.

**Conclusion:** Herein, we reported the prenatal and postnatal management for a rare case of the congenital BC by multidisciplinary approaches during the COVID-19 pandemic. Fetal MRI and screening for fetal chromosomal abnormalities are especially recommended. This case contributes to the awareness that the COVID-19 pandemic interferes with regular follow-up schedules during pregnancy and may interfere with timely performed additional tests; which leads to more accurate genetic counseling. A combination of multidisciplinary approaches, including radiology, infection control, genetic counseling, obstetrics, and pediatric surgery, is pivotal for managing fetal BC during the COVID-19 pandemic.

## Introduction

A fetal bronchogenic cyst (BC) is a benign but rare congenital anomaly and generally detected after 20 weeks of gestation by periodic prenatal checkups. Chromosomal abnormalities are considered to be associated with 1.6% of fetal pulmonary malformations. For example, there was a fetal congenital pulmonary airway malformation (CPAM) associated with mosaic Klinefelter (1). Therefore, invasive prenatal diagnosis is recommended for suspected fetal BC. However, the coronavirus disease 2019 (COVID-19) pandemic disrupted the regular schedules of most pregnant women. A case of the congenital BC was not found until 34^+6^ weeks of gestation. To improve the understanding of this rare disease, especially during the COVID-19 pandemic, we report observations and treatment by multidisciplinary approaches. This paper presents a systematic prenatal and postnatal management for fetal BCs.

## Case Description

**Figure 1:**
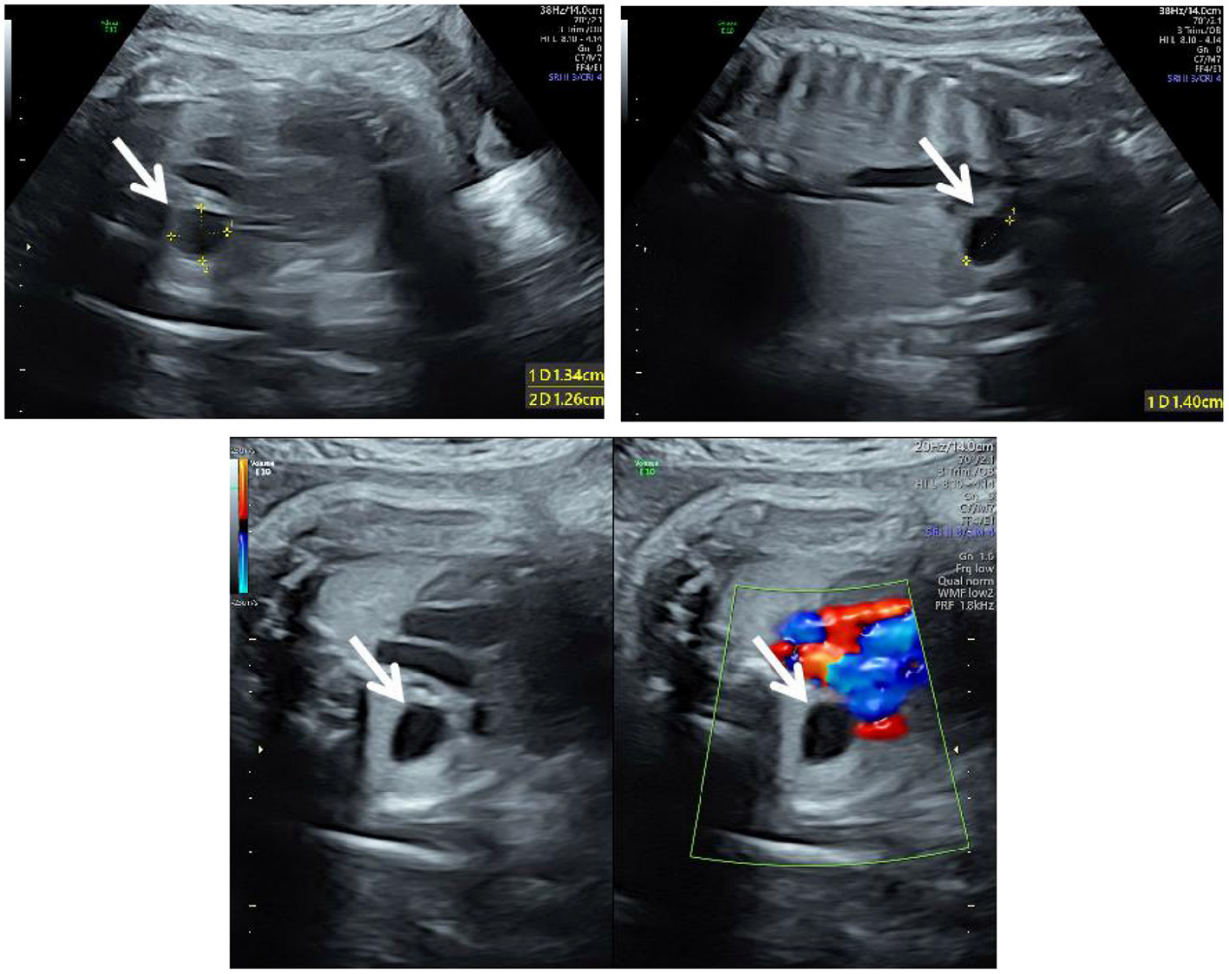
Ultrasound at 34^+6^ weeks of gestation. The three-vessel trachea section of the fetus showed an anechoic area of 1.3 × 1.4 × 1.3 cm (white arrows) on the right side of the thorax, with clear boundaries and without vascular supply; the trachea was compressed and flattened.

**Figure 2:**
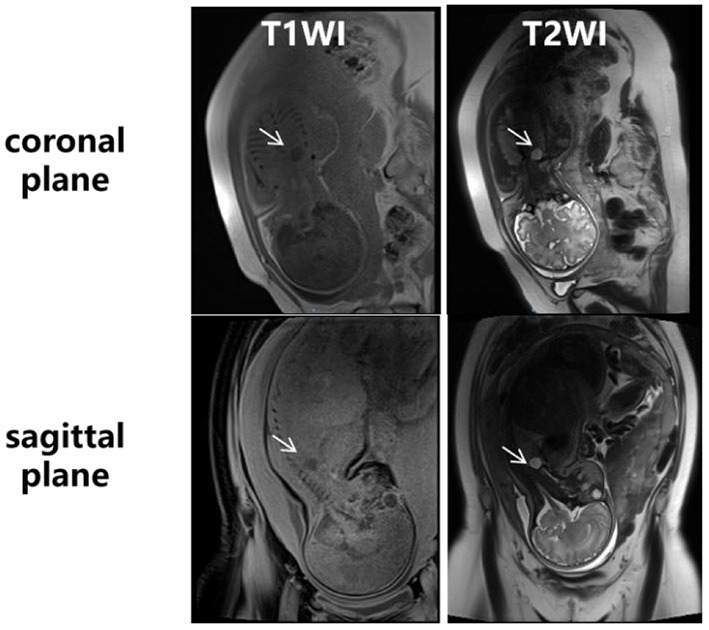
Fetal MRI at 35^+3^ weeks of gestation. An ovular-like area with long T1 and long T2 signal (white arrows) was found in the upper right thoracic cavity, on the right side of the lower trachea, above the ridge of the trachea. The boundary was clear, and the size of the lesion was about 1.5 × 1.2 × 1.5 cm.

A 31-year-old woman with a singleton pregnancy missed the regular prenatal checkups (including her second trimester ultrasound) due to the COVID-19 pandemic and came to our hospital for a fetal ultrasound examination at 34^+6^ weeks of gestation. A 1.3 × 1.4 cm cyst was found on the right side of the fetal thorax (Figure 1). Further, a fetal MRI was performed at 35^+3^ weeks of gestation. The MRI showed a 1.5 × 1.2 cm × 1.5 cm cystic lesion in the right thoracic cavity of the fetus, which was considered as a congenital BC (Figure 2). The woman was not able to undergo prenatal genetic diagnosis because of the late gestational age. A multidisciplinary consultation was implemented by the pediatric surgery, radiology, and obstetrics departments, and a regular follow-up was recommended. There was no significant change in cyst size during the follow-up. A planned cesarean section (C-section) was performed at 38^+5^ weeks of gestation due to a previous C-section and rejection of vaginal delivery. The reaction of the baby girl was good, with Apgar score 9 at 1 min and 10 at 5 min after birth.

**Figure 3:**
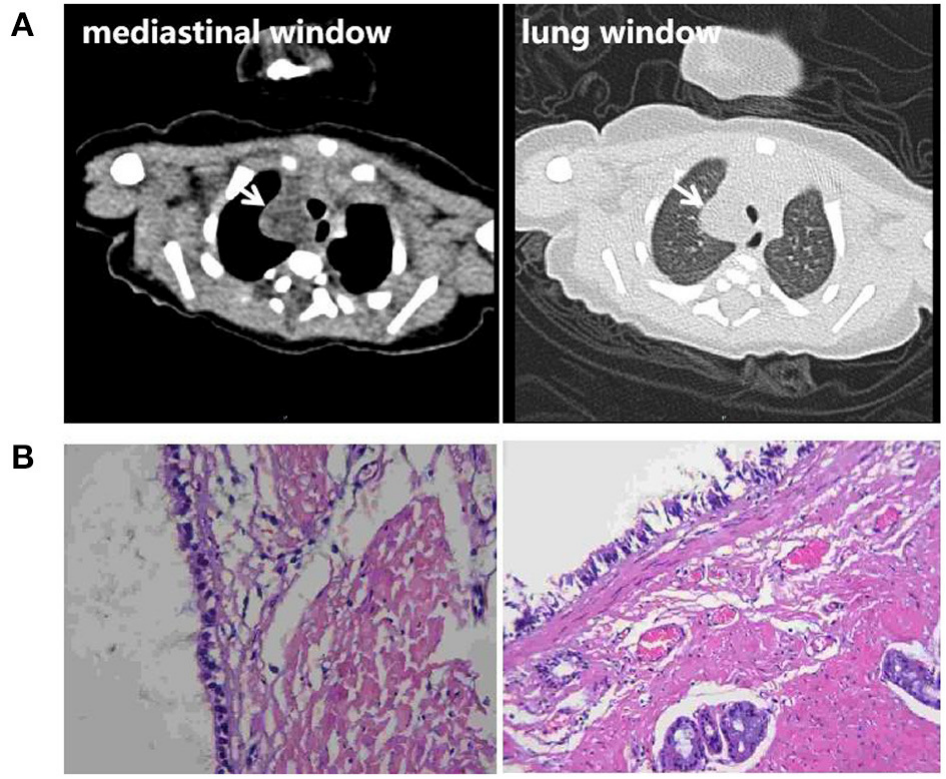
**(A)** Chest CT scan of the newborn 3 days after birth. An irregular low-density lesion with a diameter of about 16 mm was seen in the upper right mediastinum, with a slightly unclear boundary. The CT value was about 8 Hu. No abnormal thickening of the pleura was observed, and no signs of effusion were observed in the pleural cavity. **(B)** Postoperative pathological findings show a bronchogenic cyst.

A postnatal chest CT scan confirmed a cystic lesion of about 1.6 cm on the right upper mediastinum (Figure 3A). The child was breastfed and had a normal growth curve during the follow-up. A reexamination of chest enhanced CT scan was performed at 4 months of age. A 2.4 × 2.3 × 2.1 cm low-density shadow was shown on the right upper mediastinum, without enhancement after injection of a contrast agent. Considering the gradual enlargement of the cyst, the multidisciplinary team proposed a surgical treatment plan. With COVID-19 testing negative, doctors for infectious disease evaluated the risk of infection and took preventative measures during the COVID-19 pandemic. Pediatric surgeons carried out a video-assisted thoracoscopic surgery with closed thoracic drainage for the 5-month old girl. Subsequent pathological findings (Figure 3B) confirmed the diagnosis of BC. The patient's recovery was smooth, and she was discharged after 6 days. The girl recovered well with normal growth and development at present.

## Discussion

The incidence of congenital BCs was about 0.147–0.238‰; 20–30% of them are congenital anterior intestinal cysts (2, 3). By reviewing the literature, we list fetuses with BCs in Table 1. The abnormal development of the buds of the primitive foregut and tracheobronchial tree leads to the formation of BCs (8). BCs can be divided into three types according to their original location: intrapulmonary, mediastinal, and ectopic. The timing of the formation of the cyst during fetal development affects its location: If a cyst was formed in the early stage, it would more likely be mediastinal; if formed in the later stage, the cyst would more likely be intrapulmonary; if migrating farther away, the abnormal buds would be an ectopic cyst (8, 9). Most bronchial cysts are located in the mediastinum, while the ectopic cyst is exceedingly rare (10). Our reported case was a typical mediastinal BC on the right superior mediastinum.

**Table 1:** Clinical features of described fetuses with bronchogenic cysts.

**Literature**	**Gestational week**	**Cyst location**	**Complications**	**Chromosome abnormality**	**Treatment and timing**
Our case	34^+6^ weeks	In the upper right mediastinum	None	Unknown	A video-assisted thoracoscopic surgery with closed thoracic drainage, 5 months
Li et al. (4)	24 weeks	Below the tracheal carina	None	Unknown	A video-assisted thoracoscopic surgery, 6 months
Venkatesh et al. (5)	20 weeks	Omphalocele	None	Unknown	Termination of pregnancy
Chatterjee et al. (6)	21 weeks	At the level of the carina	Hyperinflation of the entire left lung and rightward mediastinal shift	Unknown	An *ex utero* intrapartum treatment (EXIT) to resection of the bronchogenic cyst *via* a fetal thoracotomy, 36 gestational weeks
Bayar et al. (7)	24 weeks	Left hemi thorax	Mediastinal shift causing decrease in lung volume	Unknown	Pulmonary drainage, every week from 24 to 30 gestational weeks

Generally, BCs have no symptoms. With the development of prenatal ultrasound and MRI, BCs can be detected prenatally after 20 weeks of gestation. The ultrasonographic characteristics of BCs are described as follows: a single hypoechoic or medium–high echoic circular or quasi-circular lesion. However, the misdiagnostic incidence of ultrasonic examination is about 25% in fetal intrathoracic malformations (11). Therefore, a fetal MRI is indispensable for further investigation of fetal pulmonary malformations. The fetal pulmonary tissue usually is a T2-hyperintense structure in MRI after 24 weeks of gestation (3). The manifestations of BCs in MRI were mainly round, well-defined cystic lesions, but a few were irregular or lobulated. Like in our case, MRI indicated classical homogeneous long T1 and long T2 signals. In 15% of cases, the BCs are parenchymatous. A differential diagnosis of CPAM, pleural pulmonary blastoma (PPB), and macrocytic lymphatic anomaly should be considered.

Chromosomal abnormalities are associated with 1.6% of fetal pulmonary malformations, especially those with pulmonary hypoplasia and bronchial cyst (12, 13). Therefore, invasive prenatal diagnosis such as amniocentesis is recommended for fetuses with BCs to exclude potential chromosomal abnormalities. Genetic counseling and multidisciplinary evaluation should be carried out. In our case, the prenatal diagnosis has not been performed because of the late gestational age. Even during the COVID-19 pandemic, a planned prenatal and postnatal follow-up is necessary.

For fetuses with BCs in prenatal MRI, according to the level of tracheal compression and pleural effusion, a multidisciplinary consultation with pediatric surgeons, radiologists, and obstetricians should be organized to discuss the fetal prognosis. The follow-up of congenital BCs may be performed every 2 weeks, depending on its location and size (3). For the fetus with a sign of compression, transfer to a fetal medicine center for further estimations and possible treatment is recommended. The mode and timing of delivery depend on the compression signs, such as fetal edema and tracheal compression. If a tracheal compression is suspected, the patient should be transferred to a tertiary hospital with a neonatal surgical team. *Ex utero* intrapartum therapy (EXIT) and extracorporeal membrane oxygenation (ECMO) should be prepared (7). In our case, the BC was relatively small with slight tracheal compression, without a sign of deterioration. A C-section was performed at term because of previous C-section and refusal of vaginal delivery.

After birth, the general condition of the neonate should be monitored, and a CT scan should be performed to estimate the size and location of the cyst, especially if there is any tracheal compression. Mediastinal bronchial cysts could occur anywhere along the mediastinum. Most mediastinal BCs are located next to the trachea and below the carina (14). The BCs mostly developed close to the trachea or bronchus but rarely communicated with the bronchus. One of the characteristic manifestations of BCs is flat borderlines with a “D” shape. The BCs are almost single, elliptic, or quasi-round, with smooth borderline and uniform foliation or soft tissue density in CT. In an enhanced CT scan, the cystic wall but not the contents would be partially enhanced. The size of the BC may gradually increase because of its secretion function. The complications include compression, infection, and hemorrhage (15). Despite the low malignancy risk, early management such as thoracoscopy for resection is recommended (4, 16, 17). For the asymptomatic patient, the timing of surgery is generally at 3–6 months after birth due to the low risk of complications (4, 18). For those with prenatal pleural effusion, a transabdominal thoracentesis or pleural amniotic shunt (TAS) could be selected to relieve the fetal symptoms after a comprehensive benefit–risk balance evaluation (3). The typical histological findings are as follows: the cyst wall is lined with pseudostratified ciliated columnar epithelium, containing airway cell components such as cartilage, glandular cells, smooth muscle, and ciliated cells (19, 20).

Most studies have proven that pregnant women are not more prone to develop COVID-19 than the general population (21). Because of the limited data on COVID-19 in the first trimester and early pregnancy, there is no scientific evidence that COVID-19 increases the risk of fetal congenital malformations or causes miscarriage in early pregnancy (22). Previous studies have not shown any detrimental effect of COVID-19 during pregnancy on fetal growth (21). Therefore, since the pregnant woman in our report did not have a COVID-19 infection, the key was to take protective measures, such as wearing a mask and refraining from going outdoors.

## Conclusions

In conclusion, a fetal MRI and planned follow-up is recommended for the fetus with a diagnosis of suspected BCs by prenatal ultrasound. Genetic counseling with fetal chromosomal analysis would be recommended for fetuses with BCs. A follow-up by a multidisciplinary team with an ultrasound assessment every 2–3 weeks will be needed. If any sign of compression shows up, an intrauterine treatment could be discussed. The mode and timing of delivery depend on the size, location, and complications of the BCs. The patient should be transferred to a tertiary hospital with a neonatal surgical team to ensure maternal–neonatal safety. For the patient with asymptomatic BCs, surgical treatment may be performed at 3–6 months after birth. The preventative measures against COVID-19 infection must be taken throughout the entire prenatal and postnatal management process.

A combination of multidisciplinary approaches, including radiology, infection, genetic counseling, obstetrics, and pediatric surgery, would provide a precise prenatal diagnosis and planned postnatal management, improving the neonatal survival rate with mediastinal masses during the COVID-19 pandemic.

## Data Availability Statement

The original contributions presented in the study are included in the article/supplementary material, further inquiries can be directed to the corresponding author/s.

## Ethics Statement

Written informed consent was obtained from the minor(s)' legal guardian/next of kin for the publication of any potentially identifiable images or data included in this article.

## Author Contributions

LC and JD collated the data, reviewed the literature, and wrote the first draft of the manuscript. MW provided substantial contribution to the drafting and critical revisions of the manuscript. DL and HL performed imaging screening for the pregnant woman and provided the photographs. JM, JL, and CW performed the consultation and followed up the fetus. YZ carried out the concepts, design of the study, approval of the final version of the manuscript, and agrees to be accountable for all aspects of the work related to accuracy and integrity. All authors read and approved the final manuscript.

## Conflict of Interest

The authors declare that the research was conducted in the absence of any commercial or financial relationships that could be construed as a potential conflict of interest.
